# Warming alters plankton body-size distributions in a large field experiment

**DOI:** 10.1038/s42003-024-07380-2

**Published:** 2025-02-03

**Authors:** Dania Albini, Emma Ransome, Alex J. Dumbrell, Samraat Pawar, Eoin J. O’Gorman, Thomas P. Smith, Thomas Bell, Michelle C. Jackson, Guy Woodward

**Affiliations:** 1 Department of Biology, University of Oxford, Oxford, UK; 2 The Georgina Mace Centre for the Living Planet, Department of Life Sciences, Imperial College London, Ascot, UK; 3 School of Life Sciences, University of Essex, Colchester, UK; 4 Somerville College, University of Oxford, Oxford, UK; 5 University of Exeter, Exeter, UK

**Keywords:** Climate-change ecology, Freshwater ecology

## Abstract

The threat of climate change has renewed interest in the responses of communities and ecosystems to warming, with changes in size spectra expected to signify fundamental shifts in the structure and dynamics of these multispecies systems. While substantial empirical evidence has accumulated in recent years on such changes, we still lack general insights due to a limited coverage of warming scenarios that span spatial and temporal scales of relevance to natural systems. We addressed this gap by conducting an extensive freshwater mesocosm experiment across 36 large field mesocosms exposed to intergenerational warming treatments of up to +8 °C above ambient levels. We found a nonlinear decrease in the overall mean body size of zooplankton with warming, with a 57% reduction at +8 °C. This pattern was broadly consistent over two tested seasons and major taxonomic groups. We also detected some breakpoints in the community-level size-temperature relationship, indicating that the system’s response shifts noticeably above a certain level of warming. These results underscore the need to capture intergenerational responses to large gradients in warming at appropriate scales in time and space in order to better understand the effects of warming on natural communities and ecosystems.

## Abstract (web summary)

Warming reduces zooplankton size by up to 57% at +8° C, with some nonlinear trends and breakpoints. This large-scale mesocosm study highlights the importance of intergenerational responses to warming for understanding ecosystem changes.

## Introduction

Understanding how climate change affects ecosystems is a critical challenge in today’s world^1^. Global warming is reshaping the properties of ecosystems and communities, changing their biodiversity, functioning and phenology^2–4^, with much of this being driven by changes in the underlying metabolism of individual organisms^5,6^. Fresh waters are especially vulnerable to climate change due to their (relatively) small size and isolation within terrestrial landscapes, which is further exacerbated by their heavy human exploitation for goods and services (e.g. over abstraction for irrigation and drinking water^7^). Long-term monitoring offers valuable insights into the past and present distributions and ecology of aquatic organisms^8^. However, predicting their responses to future climate changes remains challenging due to several factors. These include gaps in understanding the drivers of spatiotemporal changes and a lack of experimental studies conducted at relevant scales and across multiple levels of warming. Most existing studies are limited in both spatial and temporal scope and typically address only one level of warming^9,10^. Integrating analysis of functional traits alongside the more traditional analysis of taxonomic composition are also needed to reveal more general patterns across scales and levels of organisation^11,12^.

In relation to this knowledge gap, body size is a fundamental trait for predicting organismal metabolic rate and fitness, as well as a host of associated functional traits and vital rates (e.g. generation time, longevity, fecundity) that link the two, through scaling laws^13,14^. These laws also dictate that community- to ecosystem-level processes are tightly coupled to species’ body size distributions^13,15,16^. In particular, species-level size distributions can influence energy flow, nutrient cycling, and trophic interactions; for example, the presence of larger predators may exert top-down control on lower trophic levels, affecting species composition and ecosystem dynamics^17–19^. Furthermore, there is now overwhelming evidence that these size distributions for the ectothermic taxa that dominate aquatic ecosystems can shift with chronic (intergenerational) warming^20,21^, and that this is to a great extent driven by the “temperature-size rule” (TSR)^22–25^ wherein species’ mean sizes decrease non-linearly with chronic warming. The TSR is considered one of the three “universal responses” to global warming, alongside phenological and range shifts^23,26,27^. While the underlying causes of the TSR, which include decreases in carbon use efficiency due to exponentially faster growth, ontogenetic development rates at higher temperatures, are still not fully understood^28–31^, the resulting community-wide reductions in mean species’ body sizes have wide-ranging implications for ecosystem functioning (Yvon-Durocher & Allen^15^). Thus overall, the TSR, by changing species’ size distributions, can affect ecosystem functioning directly as well as indirectly through the networks of complex interactions and resulting feedback loops. These effects may be exacerbated by the fact that the TSR is typically a non-linear phenomenon wherein species’ mean size declines exponentially with temperature^15,16^.

However, despite the growing body of observational data and theory, there remains a (empirical and experimental) gap in our knowledge of the magnitude of the community- to ecosystem-scale effects of TSR across a wide spectrum of warming scenarios as well as spatial and temporal scales in natural environments^32^. Previous experimental efforts have predominantly focused on limited levels of warming above ambient conditions, typically of up to just 3–5 ˚C^21,33–35^. However, such approaches fail to capture the finer resolution along more extended temperature gradients necessary for accurately characterising the driver-response relationship and for predicting how community structure will respond to future warming. Our understanding still rests heavily on extrapolations from proxy spatial gradients, mathematical models^36^, or laboratory experiments^37,38^, rather than direct measurements from realistic large-scale and long-term field experiments encompassing multispecies systems (but see refs. ^39,40^). These limitations may be largely attributed to the logistical and financial challenges associated with implementing multiple warming levels at a scale that adequately captures responses across different biological organisation levels in semi-natural systems^41^.

Furthermore, our understanding of the effects of the TSR on vital ecosystem compartments such as planktonic communities, remains particularly poor, despite the fact that they play fundamental roles in food webs as a main source of energy and matter transfer to higher trophic levels^42^. The short generation time of plankton also means that they can respond especially rapidly to perturbations^10,43,44^. Zooplankton responses to fine resolution warming gradients are still contradictory, for instance regarding the effects on body size^45–47^. In aquatic food webs in general and planktonic food webs in particular, body size plays a critical role in determining trophic position^48–50^ and it is closely linked to various key ecological traits and vital rates, including fecundity, population growth rate, competitive interactions, population abundance, and the size distribution of biomass within the ecosystem^51,52^. Additionally, because, like most aquatic taxa, zooplankton are ectotherms, the biochemical processes and ecosystem functions they support are significantly influenced by their body size and the temperature of their environment^48,53,54^. Biochemical reactions, which are essential for processes like metabolic rates, typically increase with rising temperatures^13,55^. This temperature-induced increment of biochemical reactions is associated with a decrease in body size^7,13,23,56^. Furthermore, seasonality might play a role in the responses of zooplankton to warming. In fact, low food (i.e., phytoplankton) availability and high predator pressure in the winter and autumn season might lead to zooplankton reduced body size^57,58^. Taxonomic identity can also affect the body size responses to temperature of different zooplankton, which might be due to different food adaptability and competitive ability of different taxon groups^49^. Due to the complex interactions that plankton have across seasons and with temperature, and since zooplankton have multiple generations per year^29^, investigating responses to warming across seasons can provide important insights that cannot be obtained from laboratory studies^59^.

**Fig. 1:**
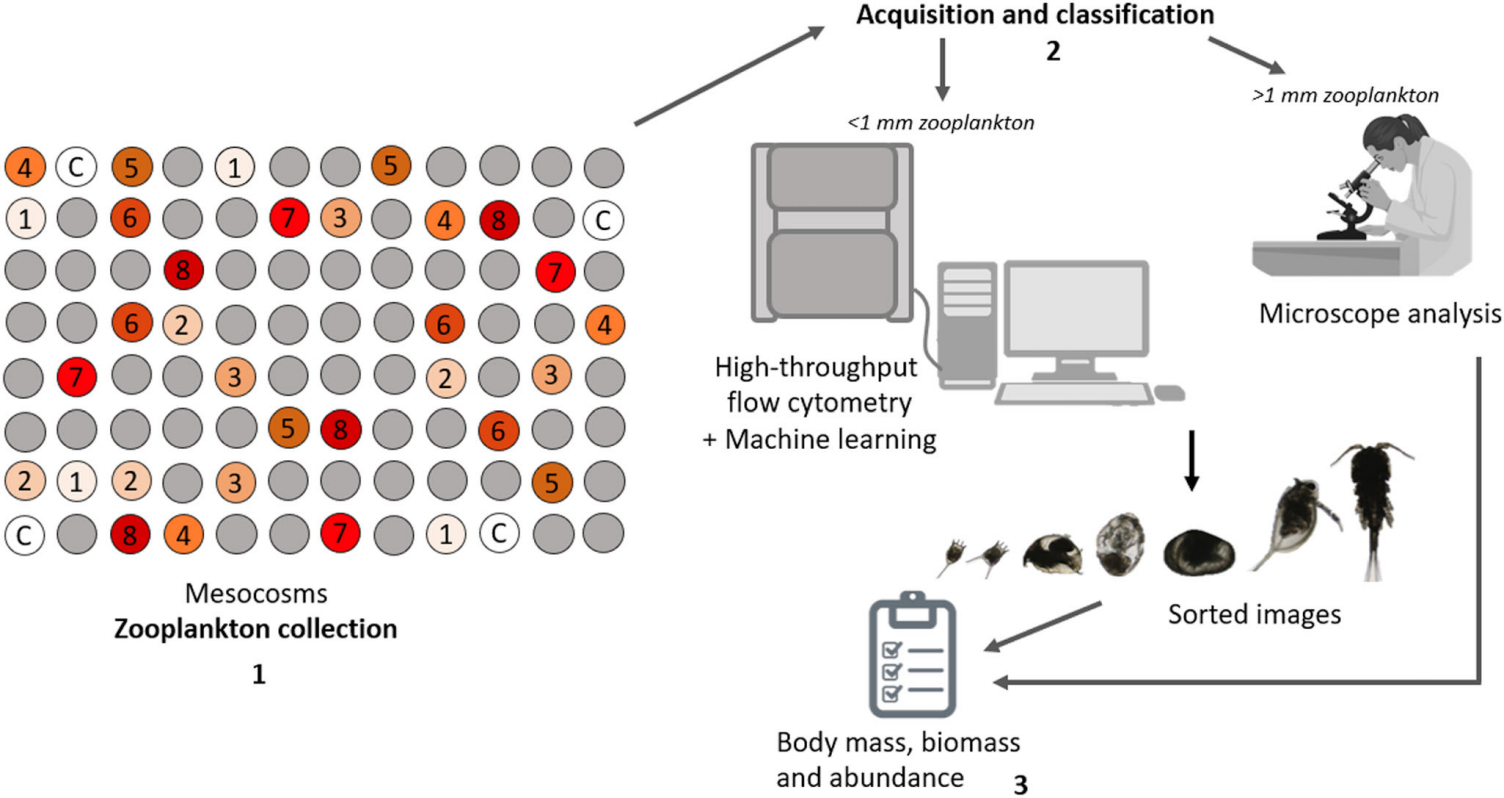
Experimental design. On the left, schematic representation of the mesocosms heated with a gradient of temperatures (from +1 to + 8 °C above ambient). Zooplankton samples were collected with a plankton net in Spring and Autumn 2019 and analysed with a FlowCam (High- throughput flow cytometry coupled with machine learning), if smaller than 1 mm, and with a microscope if bigger than 1 mm, to obtain body measurements and abundance data.

Another important aspect of the response of plankton to warming that has yet to be clarified is whether changes in body size exhibit a linear, nonlinear, or discontinuous relationship with rising temperature. Breakpoints in the interspecific, community-scale size-temperature relationship may indicate the crossing of temperature thresholds for individual species. This could be a precursor or early-warning of an imminent regime shift^60,61^ caused by changes in relative abundances of different sized species^62–65^ or alterations in predator-prey mass ratios, thereby changing community composition^66^. To address this and the gaps outlined above, we combined realistic settings of one of the largest current outdoor freshwater mesocosm arrays worldwide with high throughput automated sampling and novel data extraction tools to quantify thousands of zooplankton over two seasons, spanning multiple generations of zooplankton. Our experiment spanned 36 mesocosms subjected to 8 levels of experimental warming from 1 to 8 ^o^C above ambient conditions over two seasons (Fig. 1 and S1 in Supporting Information). We focused on 3 key questions: 1) *How does average body size change with warming*?; 2) *how do different taxonomic groups contribute to this response?*; 3) *and how consistent is the community-level response across temperatures and seasons?.*

## Results

### Q1) How does average body size change with warming?

**Fig. 2:**
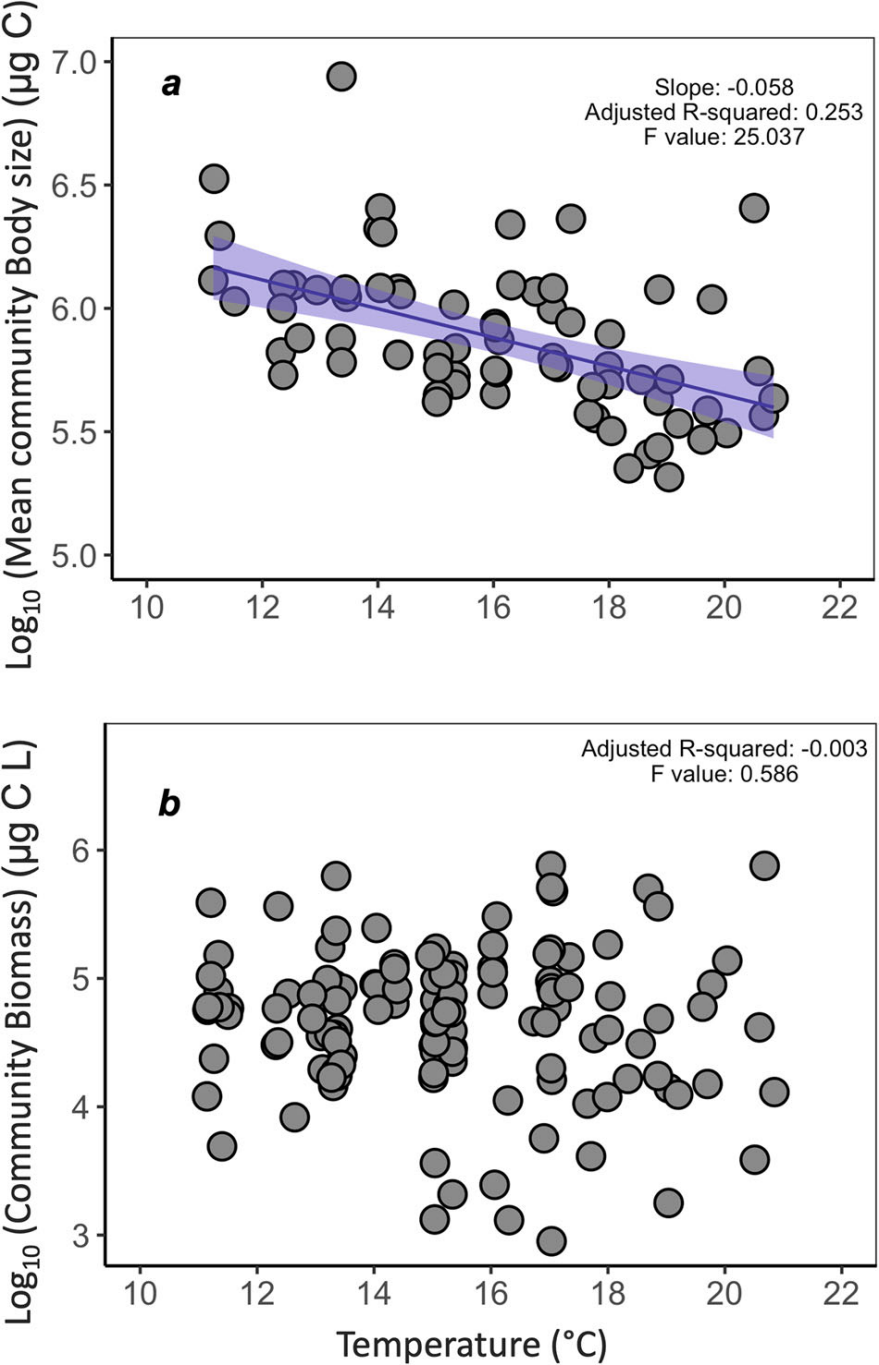
Effect of warming on community body size and biomass. **a** Effect of warming on average community Body size (y-axes, transformed in log_10_) of all measured individuals in each mesocosm (n mesocosms = 72) for each temperature (x-axes, F_1,70_ = 25.037, Adjusted R^2^ = 0.253, *p* < 0.05). Linear model is fitted showing the confidence intervals. **b** Effect of warming on the average community Biomass (y-axes, transformed in log_10_) across the mesocosms (n mesocosms = 72) for each temperature (x-axes, F_1,63.6_ = 1.1290, Adjusted R^2^ = −0.003, *p* = 0.2920).

At the community level, mean zooplankton body size declined with warming (Fig. 2a, slope = −0.058, F_1,70_ = 25.037, Adjusted R^2^ = 0.253, *p* < 0.05) but neither sampling season nor mesocosms had a significant effect (Table 1). The overall standing community biomass and abundance (except for Copepoda, whose abundance increased Figure S2) per mesocosm did not respond to warming (Fig. 2b, F_1,124_ = 0.586, Adjusted R^2^ = −0.003, *p* = 0.445).

**Table 1:** Results of segmented *vs* linear regression analysis on the influence of the gradient of temperatures on population and community zooplankton average body size

			Average population body mass					
Taxon		Selected model	Model	Breakpoint T (^o^C)	R^2^	*p*-value	Slope	CIs
Copepoda		Linear	Lm(mean_log_bodysize ~ temp,Copepoda)	/	0.248	< 0.001	−0.086	−0.12, −0.05
Ostracoda		Linear	lm(mean_log_bodysize~temp+date,Ostracoda)	/	0.611	< 0.001	−0.12 (Aut), −0.11 (Spr)	0.15 −0.09 (temp); 0.016, 0.30 (date)
*Daphnia spp*.		Segmented	segmented(Daphnia, seg.Z = ~ temp, psi =NA, control = seg.control(K = 1))	19.20	0.218	0.014	−0.068, −0.079	1) −0.12 −0.01; 2) −0.29 0.45
*Alona spp*.		Linear	Lm(mean_log_bodysize ~ temp,Alona)	/	0.755	< 0.001	−0.056	−0.07 −0.034
*Keratella quadrata*		Segmented	segmented(K_quadrata, seg.Z = ~ temp, psi =NA, control = seg.control(K = 1))	12.64	0.050	0.031	0.019, −0.008	1)−0.06, 0.10; 2) −0.02 −0.00
*Keratella cochlearis*		Linear	Lm(mean_log_bodysize ~ temp,K.cochlearis)	/	0.000	<0.001	−0.007	−0.023 0.01
*Asplanchna spp*.		Segmented	segmented(Asplanchna, seg.Z = ~ temp, psi =NA, control = seg.control(K = 1))	13.46	0.367	<0.001	−0.011, −0.040	1) −0.07 0.05; 2) −0.06 −0.02
			**Average community body mass**					
		**Selected model**		**Breakpoint T (**^**o**^**C)**	**R**^**2**^		**Slope**	
Community		Linear		/	0.253		−0.058	−0.08 −0.03

Breakpoints are given as absolute temperature. Slopes and Confidence Intervals (CI) are given for each model. When the segmented regression was the best fit, all the slopes and breakpoints are reported. *temp.* Temperature, *mean_log_bodysize* average body size, log10 transformed.

### Q2) How do different taxonomic groups contribute to this response?

**Fig. 3:**
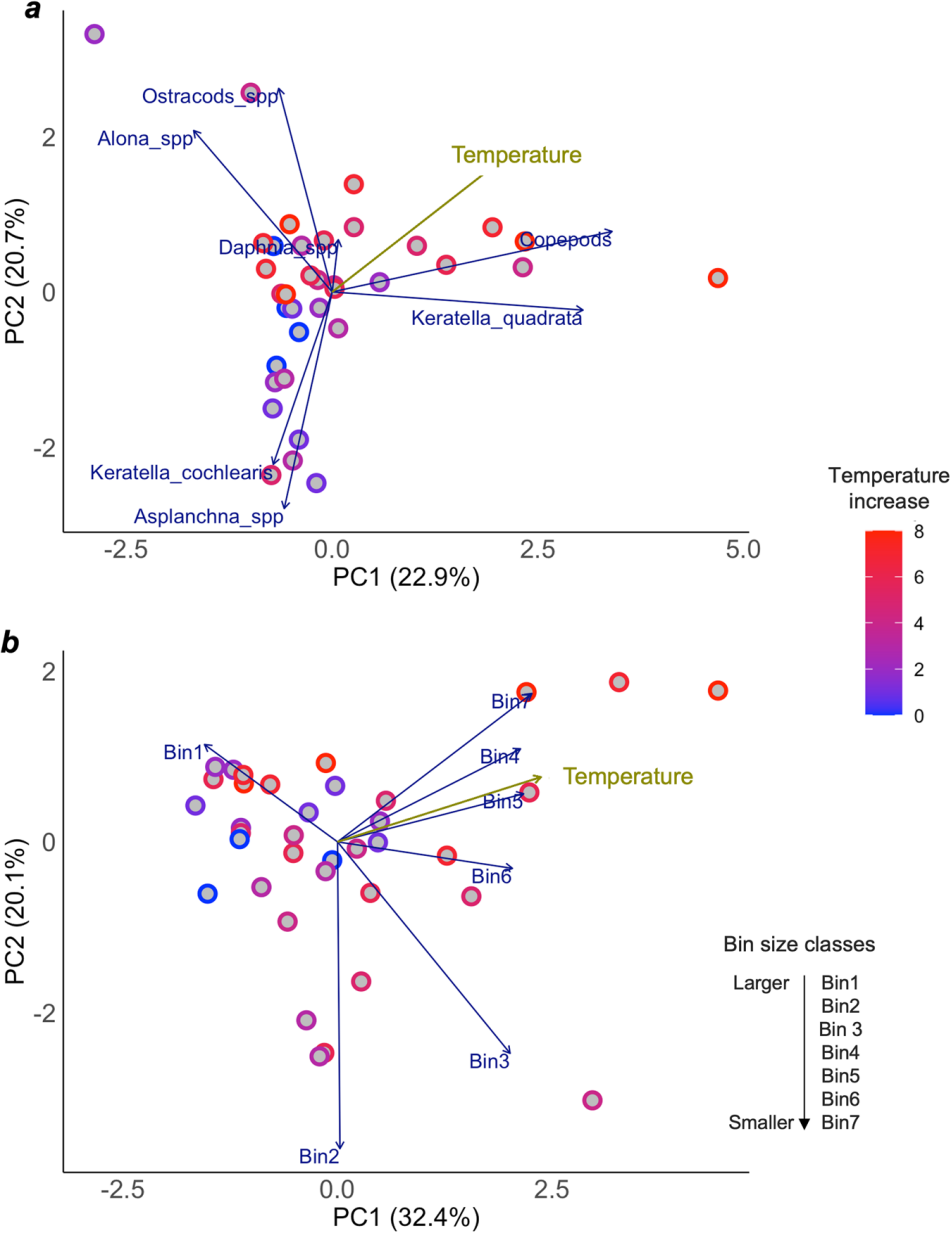
PCA Biplots of Taxa Abundance and Body Size with Temperature. **a** The biplot displays the results of principal component analysis (PCA) conducted on the taxa abundance per mesocosm averaged over the sampling times. Each point represents an individual mesocosm, and its position in the plot reflects its projection onto the first two principal components (PC1, x-axes and PC2, y-axes). The first axis explains 22.9% of the variance, while the second 20.7%. The length and direction of the arrows represent the contribution and direction of the taxa abundance to the principal components (PC1 and PC2). **b** The biplot displays the results of principal component analysis (PCA) conducted on the individual body size averaged over the sampling times. Each point represents an individual mesocosm, and its position in the plot reflects its projection onto the first two principal components (PC1, x-axes and PC2, y-axes). The first axis explains 32.4% of the variance, while the second 20.1%. The length and direction of the arrows represent the contribution to the principal components (PC1 and PC2) and direction of the abundance of individuals with body size which fell in each bin (from 1 -larger individuals, to 7- smaller individuals). For both PCAs, temperature across samples is overlayed on the graphic, represented by a brown arrow. The colour scale indicates deviations of temperature from ambient levels, ranging from 0 °C (dark blue – control mesocosms) to 8 °C (dark red), for each mesocosm.

A PCA plot of zooplankton abundance across all taxon revealed that warming influenced species composition (PC1 explains 22.9% of the variance and is correlated with temperature with *r* = 0.43), with zooplankton in warmer ponds (indicated by red shades) differing from those in cooler ponds (blue shades), as relative abundances shifted with increasing temperature (Fig. 3a).

A second PCA constructed with zooplankton body size measurements configured into size classes from each mesocosm, shows an even clearer separation of temperature treatments, with samples with predominance of the larger bins (i.e., bins 1, 2, and 3) versus those with smaller organisms (i.e., bins 5, 6, and 7). Where the samples with larger organisms tended to correspond to lower temperature treatments (mesocosms in in blue shades), and the opposite for those mesocosms subjected to higher temperatures (mesocosms in red shades) (Fig. 3b). PCA 1 explained 32.4% of the variance (with a temperature correlation of *r* = 0.47), while PCA 2 explained a further 20.1%.

**Fig. 4:**
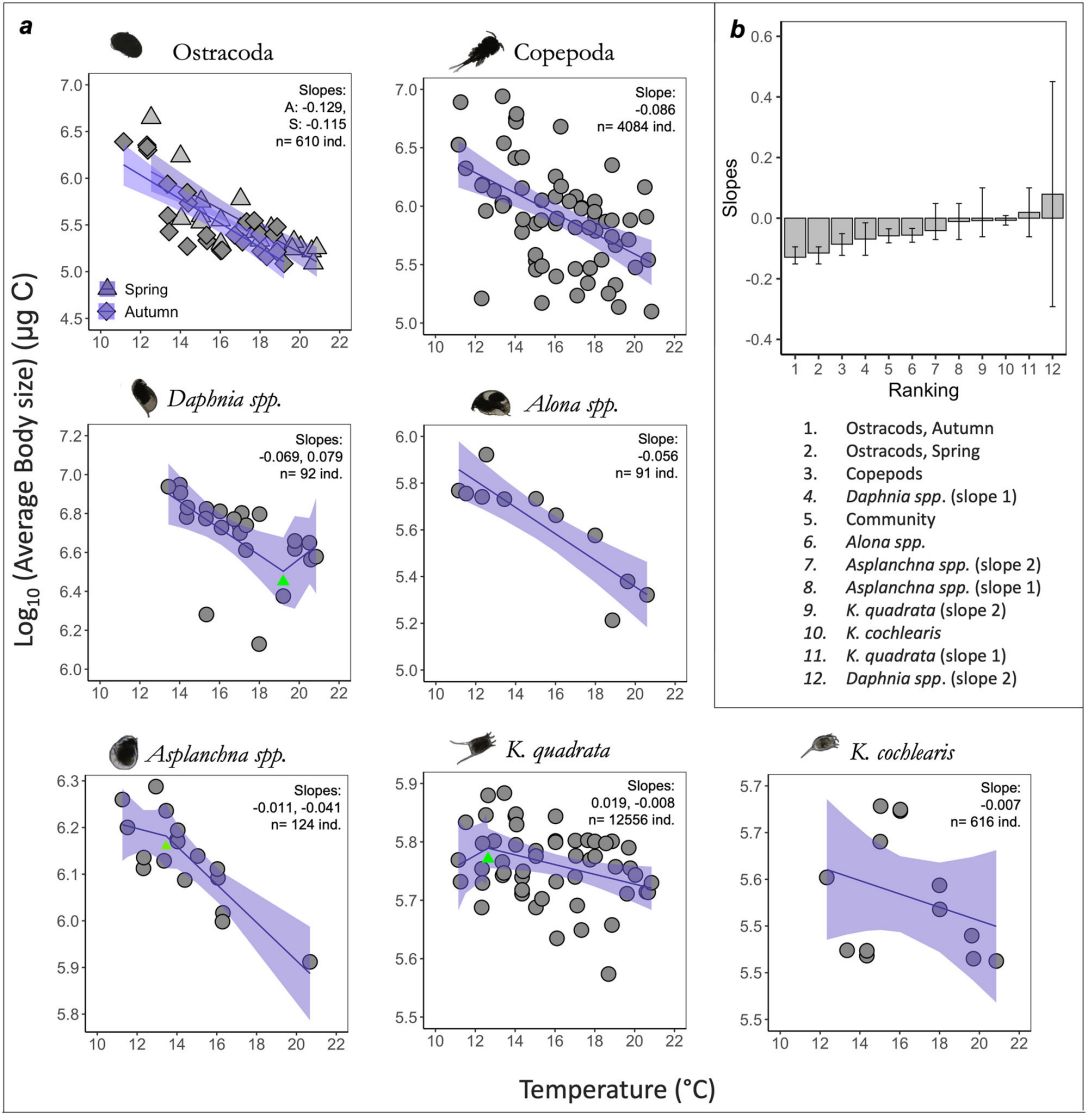
Zooplankton Body Mass *vs* Warming. **a** Relationships between warming (x-axes, in C) and average body mass (y axes, mean values in log_10_ scale, expressed in units of carbon (µg C)) in the different zooplankton taxa (total n of zooplankton = 18174 ind.). The best fitted model between segmented regression and linear model was chosen based on the AIC criterion. For each model, number of individuals and slope values are indicated for each taxon and when the segmented model was the best fit, a green triangle marks the position of the threshold points. **b** Slopes of the model for the different taxa, ranked from the steepest to the gentler slope. The shadow around the slope corresponds to the 95% confidence intervals.

At the population level, the body size of most taxa declined with warming, and this was especially marked among the three largest taxa - the Ostracoda, Copepoda and *Daphnia* spp (Table 1, Fig. 4).

### Q3) How consistent is the community-scale response across temperatures and seasons?

Among the seven taxa detected, only three – *Keratella quadrata*, *Daphnia* sp. and *Asplanchna* spp. – had their responses to warming better described by a segmented regression than by a single regression, (lower AIC in Table S1). These three taxa showed a single breakpoint each. For *K. quadrata* and *Asplanchna* spp., those breakpoints (at 12.6 ^o^C and 13.4 ^o^C respectively) corresponded to the point where the body size decreased more rapidly. In contrast, for *Daphnia* spp., the breakpoint, which occurred at 19.2 °C, was followed by an increase in the recorded body size (Fig. 4a, Table 1). The season of sampling did not exert a significant influence, either independently or in interaction with warming, on the reduction in zooplankton body size. This lack of impact was observed at community level and across all taxon populations, with the exception of Ostracoda which exhibited a steeper decrease with temperature in autumn (Table 1, Figs. 2, 4).

## Discussion

Rapid global change has renewed calls for more realistic and biologically complex ecological experiments across fine-resolution temperature gradients^67,68^ rather than the typical binary control-impact design with a single level of warming. We did this for the first time, to our knowledge, using a finely resolved thermal gradient in a large-scale freshwater mesocosm experiment and found that warming did indeed favour the small. This effect was observed both at the community level and among individual zooplankton taxa. Our findings carry significant implications for understanding how various degrees of global warming will affect aquatic ecosystems. Furthermore, we showed that some taxa responses were not monotonic, implying that complex and varied responses can emerge, especially in multispecies ecosystems. This highlights the importance of our advanced experimental design, which goes beyond binary temperature comparisons and limited thermal gradients, providing a more nuanced prediction of the effects of future climate change.

Warming significantly reduced the average body mass of zooplankton at community (Fig. 2a) and population (Fig. 4, Table 1) levels, supporting findings from previous studies using much more limited temperature gradients^69,70^. The decrease of zooplankton body size with warming could be caused by various mechanisms, such as the rising metabolic demands^13,55,71^ needed to maintain basal (i.e., maintenance) and active (e.g., reproduction and growth) metabolism, or by a thermally-induced increase in oxygen demand that exceeds the available rate of supply^72^. Assuming all else is equal (e.g., quantity and/or quality of food^73^), warming should favour the small. Our results support this theory, which will have further implications for nutrient cycling and other associated ecosystem processes^74,75^. Other mechanisms at the base of those changes can be density-dependent growth, size-dependent survival, and size-selective predation^55,61,76,77^. Future work should focus on conducting long-term experiments encompassing a wide range of thermal gradients to further investigate the complex interactions driving the observed decrease in zooplankton body size with warming, using, for instance, in-situ mesocosms.

Most taxon population responses to warming were best described by a simple linear model (Fig. 4a, Table 1), supporting other recent studies over limited thermal gradients^78^. We found steeper slopes (and therefore bigger reductions in size with each degree of warming) for Ostracoda, Copepoda and *Daphnia* spp. (Fig. 4b); the three larger zooplankton taxa we found in our mesocosms. This could be because those are the larger species, who might need to reach a smaller body size faster than already smaller-sized organisms to succeed in a warmer environment. The segmented (non-linear) model was favoured for responses exhibited only by *K. quadrata*, *Asplanchna* sp and *Daphnia* spp. This might be due to heterogeneous changes across different components of those groups, such as species turnover and relative abundance^62–65,79^). Specifically, we observed an increase in body size in *Daphnia* spp. after the temperature breakpoint. This could be attributed to the potential replacement of smaller *Daphnia* species with larger ones once the temperature reached the breakpoint. Unfortunately, we were unable to test this hypothesis with our quantification method (i.e., FlowCam). In contrast, *K. quadrata* and *Asplanchna* sp. showed a steeper decrease in body size beyond their respective breakpoints (12.6 ˚C and 13.5 ˚C) compared with *Daphnia* spp., which had a higher breakpoint (19.2 ˚C), indicating that those organisms might have a narrower thermal tolerance range compared to the other taxa. Once the temperature exceeds a certain limit, these species could experience more severe negative effects that accelerate the decrease in body size^61^. Breakpoints and non-linearities should be considered when predicting the impacts of climate change, as rates and direction of change are likely to alter with warming, and a small increase in temperature could have substantial impacts on aquatic organisms.

At the community level, mean zooplankton body size across each mesocosm was best described by a linear model, where temperature was the only factor affecting the change in body size (Table 1, Figs. 2a, 3). This could be explained by the fact that of the seven taxa detected, only three showed non-linearities, and thus the cumulative effect at the community level was a general linear response. This implies that if we only consider one level of organisation, such as the community level response here, we might miss important trends happening at other levels. Overlooking these trends could be important, as changes in individual taxa can have cascading effects throughout the ecosystem, with consequences for biodiversity, trophic interactions, and ecosystem functioning. The slope of body size decrease at the community level was higher than the slope for most of the taxa found, as it ranked 5^th^ out of the 12 slopes we found. This overall pattern could be driven by the sharp decline in body size among the three larger species, underscoring the influence of specific taxa on the community’s aggregate response.

Community biomass (Fig. 2b) and abundance (Figure S2) per mesocosm were not significantly affected by warming, which suggest compensatory effects from heat-tolerant species. Alternatively, external factors not accounted for in this study might explain this result. For instance, Yvon Durocher et al. ^47^ found a similar result with 4 ˚C of warming, attributing it to a substantial decrease in total phytoplankton biomass (i.e., the main food source for zooplankton) in the warmed mesocosms. Other studies have shown that the population stability of dominant species strongly influences community biomass stability, especially when communities are dominated by a few species^80,81^. However, our PCA plot (Fig. 3) showed that warming influences the species compositional patterns when considering their abundance. Hence, if climate change alters species asynchrony and/or the stability of dominant species, this may play a role in altering community biomass.

In our experiment, the season of sampling did not influence the decrease of body size or biomass of zooplankton. Previous research on warming in mesocosms has shown contrasting results, with some studies supporting a lack of seasonal influence^47,82,83^, while others have found seasonal responses^21^.The extended timescale of our sampling suggests that these observed body size reductions are chronic rather than transient, reflecting longer-term effects rather than short-term responses. This implies that the changes we are observing are likely enduring, rather than temporary shifts.

Our finding of an overall monotonic reduction in the body size of zooplankton at both taxon population and community levels across a range of temperatures confirms that freshwater ecosystems might be more susceptible to future warming than previously anticipated. This is noteworthy because much of our existing knowledge is derived from experimental studies that involve only limited temperature ranges (e.g., control vs 1 or 2 temperature increases) or from theoretical work that focuses mainly on linear responses. Our research demonstrates that employing a gradient-based approach allows us to capture nonlinear effects of warming in increasingly threatened freshwater habitats. Small-scale laboratory experiments testing a limited temperature range may not effectively detect such changes. Therefore, we propose a reassessment of previous predictions, which often rely on a consistent trajectory devoid of changing slopes across the environmental gradient. It is important to consider the rise of potential breakpoints and nonlinearities to advance our comprehension of both current and future climate change responses. Identifying general patterns and developing a mechanistic understanding will require a new generation of similarly large-scale experiments that can unravel the full range of impacts across multiple levels ecological organisation, from individuals to entire ecosystems, and that also span broad thermal gradients associated with predicted future warming scenarios.

## Methods

### Experimental design

This study was performed at the Silwood Park Mesocosm Facility in south-east England (Lat:51.410250 Long: −0.638620, Ascot, UK, Fig. S1), as part of a long-term warming experiment. Ninety-six 2000L mesocosms (1.0 m deep x 1.6 m wide), arranged in a 12 × 8 grid within which treatments were assigned at random within four blocks (3 × 8 grids), were seeded from a “common garden” of organisms collected from the regional species pool of fresh waters within a 5 km radius in spring 2016. The mesocosms were then left to establish as replicate communities until September 2018, at which point 32 of the 96 mesocosms were heated in 8 replicated (*n* = 4) experimental warming treatments from 1 to 8 °C above ambient water temperature in 1 °C increments. We randomly selected 4 further unheated mesocosms as controls to obtain a balanced design across the full thermal gradient, with 4 replicates each from the ambient control ponds and each of the 8 warming treatments (Fig. 1). Warming was achieved using custom-made immersion heating elements (700 W; Jevi) based on those used in our previous experiments^20,39,47^. Two high-resolution thermistors and a CR6 series data logger (Campbell Scientific) measured tank temperature, controlled by a Solid-State relay which activated the heating element, and which communicated with the other data loggers in the array. This enabled us to derive and control the daily mean temperature across all ambient ponds, every 5 minutes, to then set the real-time temperature differential for warming each heated mesocosm.

### Zooplankton sampling, enumeration, and body size measurements

Zooplankton communities from each of the studied mesocosms were sampled in Spring and Autumn 2019, respectively. Zooplankton communities were collected from the top 20 cm of each mesocosm using a 50 um mesh plankton net with two perpendicular drags across the entire diameter, resulting in a total filtered volume of 43.92 L. Once in the laboratory, we identified the primary zooplankton taxa, analysing subsamples under a dissection microscope (Leica S9E) at 50× magnification and a Zeiss Compound Microscope at 100 × and 200 ×, using published taxonomic keys^84^ (MRC Technical Paper No.45 2015). Subsequently, we processed zooplankton samples by sorting them into three size classes: 75–250 µm, 250–1000 µm and > 1000 µm), using different sieves. These samples were then preserved in 70% Ethanol for a final volume of 50 mL, in preparation for measurement and enumeration. A sub-sample of 20 mL was used to perform the image analysis for all the size classes. The first two zooplankton size classes (75–250 µm, 250–1000 µm) were analysed using a Bench Top FlowCAM^®^ 8000 (Fluid Imaging Technologies, Inc. Maine, USA) with particle analysis software (VisualSpreadsheet^©^, version 4). For these size classes, data acquisition was performed in the AutoImage mode, capturing each particle image at a user-defined rate and flow (Fluid Imaging Technologies Inc. 2011). For the smaller zooplankton in the 75-250 µm size range, we used a 4× objective lens, a Field of View 300 flow cell (with dimension of 300 µm depth, 3000 µm width), an imaging rate of 18 frames per second, and a flow rate of 2 ml/min. For zooplankton measuring between 250 and 1000 µm, we used a 2× objective lens, a Field of View 1000 flow cell (1000 µm depth, 3000 µm width), an imaging rate of 5 frames/second, and a flow rate of 6 ml/min. A small number of organisms fall into the larger class of > 1000 µm (*n* = 90). Those were analysed under a Leica S9E dissection microscope at 50× magnification, since they were too large for the FlowCam® (Method, Supplementary material). Image libraries of zooplankton categorised by species level and taxa level (when not possible to clearly identify the species with the automated method) were created prior to the experiment and were used as a reference for the auto-categorization of organisms processed. A minimum of 50 images were used to build each library. Once the sample was photographed, images were auto-identified by the VisualSpreadsheet^©^ software and classified by taxonomic groups. Manual post-processing control was performed to ensure the taxonomic sorting accuracy of the software. Once images were sorted per taxon, the total number of images per taxa was recorded and size measurements were taken. Body length and width were used to calculate of zooplankton biovolumes by assigning each organism to geometric shapes that best represented their form (i.e., rotifers: cylinder, Ostracoda: prolate spheroid, Copepoda and Cladocera: ellipsoid^85^). Biovolumes were then converted to fresh weight using a standard conversion factor of 1.1. The carbon content was estimated from a dry/wet weight ratio of 0.25^20,86^. Body mass was expressed in carbon units (µg C), assuming that dry carbon content for each zooplankton represented 40 per cent of the total dry weight^86^. Total community biomass (mg C L^−^^1^) was calculated by summing the individual body masses (mg C) per sample and dividing the volume of filtered water (L)^21^.

### Statistical analysis

All analyses were performed in R studio (version 4.2.2)^87^. Body mass and total biomass measurements were log10-transformed to meet the assumptions of normality for model residuals. Visualisations of results were performed using the package “*ggplot2*”^88^ (version 3.4.2).

We constructed a series of linear models to evaluate how warming and the month of sampling affect zooplankton body size at both the taxon population level (i.e., within taxa) and the community level (i.e., across taxa). We also analysed their impact on abundance and biomass, with all variables log_10_-transformed. We used the Akaike information criterion (AIC) to identify the most suitable model for our data. Initially, we created three models: the first included only temperature (i.e., the recorded temperature in each mesocosm at the sampling point time, ranging from an increase of +1 to +8 °C above ambient, plus the control ambient temperatures) as a factor, the second incorporated the sampling month (i.e., Spring and Autumn) as an additional factor, and the third considered the interaction between these two factors. Additionally, to account for any potential variability introduced by the mesocosms, we developed two more models: one including all three factors together and another with only temperature increase and mesocosms. Next, we used the best-fitting linear model to construct a segmented regression model (using the R package “*segmented*” version 1.6.4)^89^ to investigate temperature breakpoints. Segmented regression is a statistically robust method for detecting thresholds, determining points where the best-fit lines have significantly different slopes. This approach provides estimates of threshold values and the overall shape of the relationship^90^. We used the Akaike information criterion (AIC) to select the best model fit between the linear and segmented models (best fit model: more than 2 AIC units lower than the others^91^. For each segmented model, we calculated the slope of the segments, the confidence intervals and identified all possible breakpoints.

To further visualise the main pattern that emerged and quantify the compositional differences between treatments, an unconstrained ordination (Principal Component Analysis, PCA) was used. PCAs were performed using the temperature increase values and the community zooplankton average body size (columns) for each mesocosm (rows), using the *“factoextra”* package^92^. The Pearson correlation coefficient for temperature was calculated using the “*Hmisc”* package to show the strength and direction of the relationship between temperature and the community zooplankton average body size. A second PCA was built using the individual zooplankton body size in each mesocosm, binning the body size in 7 same-sized bin classes, following the method in Yvon-Durocher et al. ^20^.

### Reporting summary

Further information on research design is available in the Nature Portfolio Reporting Summary linked to this article.

## Supplementary information

Supplemental Material

Reporting summary

## Data Availability

The experimental data are available at the following link: 10.6084/m9.figshare.27852639^93^.

## Code availability

The R codes are available at the following link: 10.6084/m9.figshare.27852639 (Albini et al.^93^).
