# Supplementary material for: Warming alters plankton body-size distributions in a large field experiment
Source: Commun Biol. 2025 Feb 3;8:162. doi: 10.1038/s42003-024-07380-2 (PMC11790927; doi:10.1038/s42003-024-07380-2)
Supplement: Supplementary file 1 — Supplemental Material [file 42003_2024_7380_MOESM1_ESM.pdf]

<u>Supplementary material for the manuscript:</u> Warming alters plankton body-size distributions in a large field experiment , by Albini et al. 2024

FlowCam methods

FlowCAM® 8000 uses a combination of imaging, laser light and machine learning to quickly detect particles within a fluid sample, enabling the capture of particle images for semi-automatic image analysis. FlowCAM® 8000 combines the technologies of flow cytometry, microscopy and image analysis (Sieracki et al. 1998). The instrument works as follows: particles contained in a water sample are drawn from an inlet port through a glass flow chamber by a peristaltic syringe pump. As particles pass through the flow chamber, they are illuminated by a laser and a digital image is captured for each single particle, by a digital camera and the images are collected and stored in a computer. The FlowCAM is provided with different microscope lenses, flow chambers and syringe pumps based on the size of the particles to be analysed (Sieracki et al. 1998, Álvarez et al. 2012), for a minimum of 20 µm and a maximum size of 1000 µm.

Figures

Figure S1: Mesocosms facilities at Silwood Park, Imperial College, UK

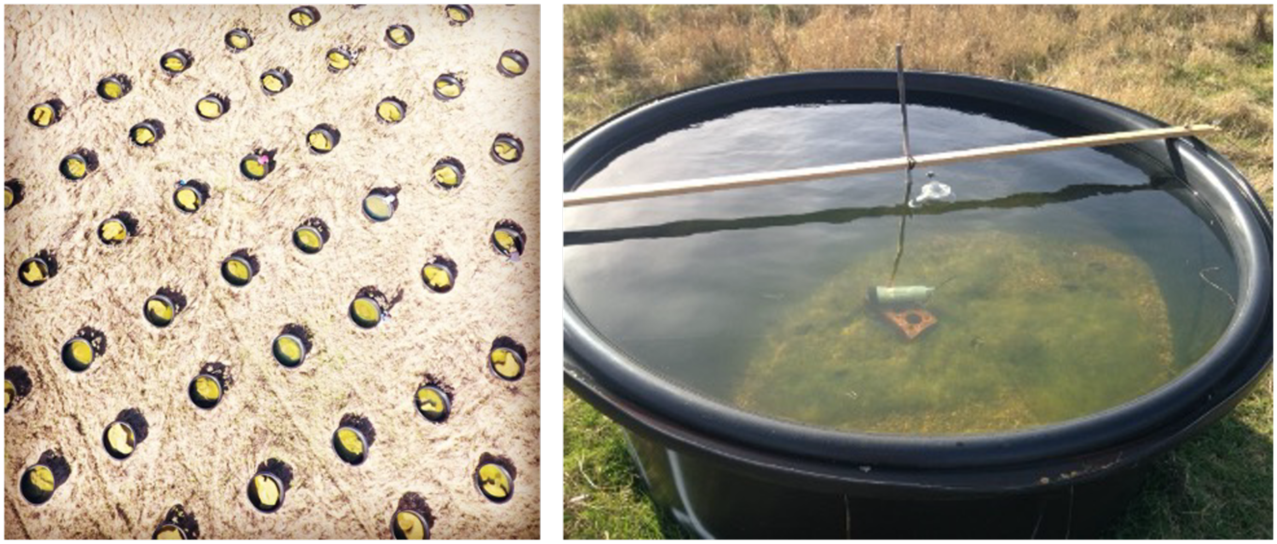

Figure S2: Y-axes shows the changes in population taxa abundance (Individuals/L+1), with the increase of temperature (x-axes).

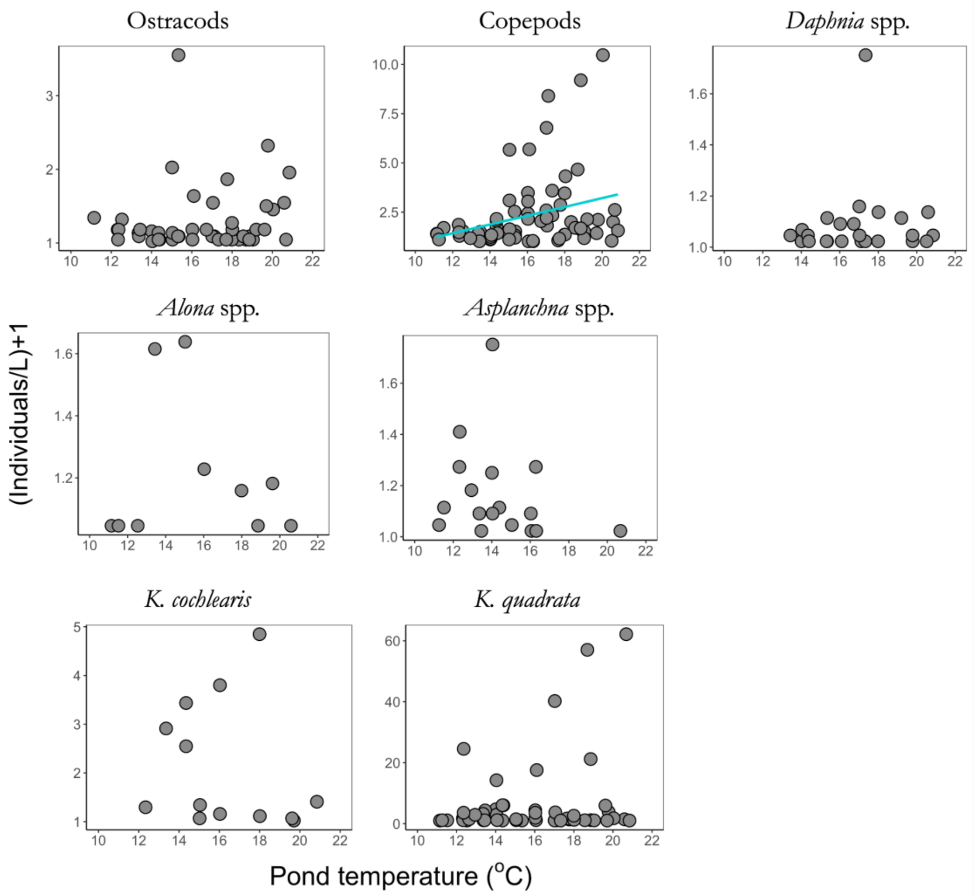

Table S1. AIC values for the linear model that best fit our data (out of the 5 models compared – see method and Table 1 for the description of the models) vs the segmented model

Taxon	***Selected model***	***AIC linear vs segmented model***
Copepoda	Linear	67.16 *vs* 69.15
Ostracoda	Linear	0.63 *vs* 21.18
Daphnia spp.	Segmented	11.74 *vs* 9.89
Alona spp.	Linear	13.69 *vs* 13.69
Keratella quadrata	Segmented	141.86 *vs* 138.74
Keratella cochlearis	Linear	29.95 *vs* 34.86
Asplanchna spp.	Segmented	45.71 *vs* 43.29
