# Supplementary material for: Warming alters plankton body-size distributions in a large field experiment
Source: Commun Biol. 2025 Feb 3;8:162. doi: 10.1038/s42003-024-07380-2 (PMC11790927; doi:10.1038/s42003-024-07380-2)
Supplement: Supplementary file 2 — Reporting summary [file 42003_2024_7380_MOESM2_ESM.pdf]

nature portfolio

Corresponding author(s): Dr. Dania Albini

Last updated by author(s): 15/11/24

Reporting Summary

Nature Portfolio wishes to improve the reproducibility of the work that we publish. This form provides structure for consistency and transparency in reporting. For further information on Nature Portfolio policies, see our Editorial Policies and the Editorial Policy Checklist.

Statistics

For all statistical analyses, confirm that the following items are present in the figure legend, table legend, main text, or Methods section.

n/a	Confirmed	
☐	☒	The exact sample size (n) for each experimental group/condition, given as a discrete number and unit of measurement
☐	☒	A statement on whether measurements were taken from distinct samples or whether the same sample was measured repeatedly
☐	☒	The statistical test(s) used AND whether they are one- or two-sided<br>*Only common tests should be described solely by name; describe more complex techniques in the Methods section.*
☐	☒	A description of all covariates tested
☐	☒	A description of any assumptions or corrections, such as tests of normality and adjustment for multiple comparisons
☐	☒	A full description of the statistical parameters including central tendency (e.g. means) or other basic estimates (e.g. regression coefficient) AND variation (e.g. standard deviation) or associated estimates of uncertainty (e.g. confidence intervals)
☐	☒	For null hypothesis testing, the test statistic (e.g. F, t, r) with confidence intervals, effect sizes, degrees of freedom and P value noted<br>*Give P values as exact values whenever suitable.*
☒	☐	For Bayesian analysis, information on the choice of priors and Markov chain Monte Carlo settings
☒	☐	For hierarchical and complex designs, identification of the appropriate level for tests and full reporting of outcomes
☒	☐	Estimates of effect sizes (e.g. Cohen's d, Pearson's r), indicating how they were calculated

Our web collection on statistics for biologists contains articles on many of the points above.

Software and code

Policy information about availability of computer code

Data collection	no software was used to collect the data
Data analysis	we used R studio to perform statistical analysis and to create all the graphs

For manuscripts utilizing custom algorithms or software that are central to the research but not yet described in published literature, software must be made available to editors and reviewers. We strongly encourage code deposition in a community repository (e.g. GitHub). See the Nature Portfolio guidelines for submitting code & software for further information.

Data

Policy information about availability of data

All manuscripts must include a data availability statement. This statement should provide the following information, where applicable:

- Accession codes, unique identifiers, or web links for publicly available datasets
- A description of any restrictions on data availability
- For clinical datasets or third party data, please ensure that the statement adheres to our policy

the dataset and the codes used to perform the statistical analysis and to create the graphs will be available as soon as the manuscript will be accepted

Research involving human participants, their data, or biological material

Policy information about studies with human participants or human data. See also policy information about sex, gender (identity/presentation), and sexual orientation and race, ethnicity and racism.

Reporting on sex and gender	na
Reporting on race, ethnicity, or other socially relevant groupings	na
Population characteristics	na
Recruitment	na
Ethics oversight	na

Note that full information on the approval of the study protocol must also be provided in the manuscript.

Field-specific reporting

Please select the one below that is the best fit for your research. If you are not sure, read the appropriate sections before making your selection.

☐ Life sciences ☐ Behavioural & social sciences ☒ Ecological, evolutionary & environmental sciences

For a reference copy of the document with all sections, see nature.com/documents/nr-reporting-summary-flat.pdf

Ecological, evolutionary & environmental sciences study design

All studies must disclose on these points even when the disclosure is negative.

Study description	We conducted a freshwater mesocosm experiment, to test for thermal breakpoints in freshwater zooplankton comminities. Mesocosms were warmed incrementally up to 8°C above ambient, and changes in zooplankton body size, abundance, and biomass were tested. We used 4 replicates per temperature.
Research sample	We used freshwater zooplankton as model organisms. Planktonic communities are ideally suited for investigating warming impacts in aquatic ecosystems, because they have short lifespans, multiple generations a year, respond quickly to perturbations, and play fundamental roles in food webs as the main transfer of energy and carbon to higher trophic levels. Moreover, In planktonic food webs, body size is the main determinant of trophic position and is correlated with traits associated with the fitness and physiology of individual organisms, through to higher-level characteristics such as population abundance and biomass size-spectra. Furthemore, because zooplankton are ectotherms, their metabolic rates and the ecosystem processes they underpin will be driven largely by their body size and the temperature of their environment.
Sampling strategy	All mesocosms across the temperature gradient were sampled, except where more than 4 replicates were available. Here, 4 mesocosms were randomly chosen.
Data collection	Zooplankton communities were collected from the top 20cm of each mesocosm using a 50um mesh plankton net with two perpendicular drags across the entire diameter, resulting in a total filtered volume of 43.92 L. Dr. Michelle Jackson and other members of the Imperial College of London Bioscience Department helped with the sampling collection.
Timing and spatial scale	Mesocosms were sampled twice: in Autumn (October 2019) and in Spring (April 2019)
Data exclusions	no data were excluded from the analysis
Reproducibility	The experiment was not repeated – it is a long term (years) mesocosm experiment.
Randomization	After mesocosms were established, treatments were randomly assigned to them to avoid bias.
Blinding	NA

Did the study involve field work? ☒ Yes ☐ No

Field work, collection and transport

Field conditions	Mesocosms were built in a field near Silwood Park (Imperial College of London)

Location	Silwood Park, Imperial College of London
Access & import/export	NA
Disturbance	No disturbance was created

Reporting for specific materials, systems and methods

We require information from authors about some types of materials, experimental systems and methods used in many studies. Here, indicate whether each material, system or method listed is relevant to your study. If you are not sure if a list item applies to your research, read the appropriate section before selecting a response.

Materials & experimental systems

n/a	Involved in the study	
☒	☐	Antibodies
☒	☐	Eukaryotic cell lines
☒	☐	Palaeontology and archaeology
☒	☐	Animals and other organisms
☒	☐	Clinical data
☒	☐	Dual use research of concern
☒	☐	Plants

Methods

n/a	Involved in the study	
☒	☐	ChIP-seq
☒	☐	Flow cytometry
☒	☐	MRI-based neuroimaging
